# Reduced Opioid Consumption with Pericapsular Nerve Group Block for Hip Surgery: A Randomized, Double-Blind, Placebo-Controlled Trial

**DOI:** 10.1155/2022/6022380

**Published:** 2022-12-15

**Authors:** Chan Jong Chung, Deuk Won Eom, Tae Young Lee, Sang Yoong Park

**Affiliations:** Department of Anesthesiology and Pain Medicine, Dong-A University College of Medicine, Busan, Republic of Korea

## Abstract

The pericapsular nerve group (PENG) block targets the nerves innervating the anterior hip surface; however, few studies on this technique are currently available. We investigated the effects of the PENG block on postoperative opioid consumption after a hip surgery. This was a randomized, double-blind, placebo-controlled study conducted at a single institution. Fifty patients undergoing hip surgery were randomly allocated, 25 in each group, to receive a PENG block either using 25 mL of 0.5% ropivacaine (PENG group) or 25 mL of saline (control group). The primary outcome was the total opioid consumption 24 h postoperatively. The secondary outcomes were postoperative pain scores, time to first opioid demand, sensory block efficiency, quadriceps muscle strength, complications, and patient satisfaction. Compared with those in the control group, patients in the PENG group had a significantly lower total opioid consumption 24 h postoperatively (440.72 ± 242.20 *μ*g vs. 611.07 ± 313.89 *μ*g, *P* = 0.037) and significantly lower pain scores at 30 min postblock and 6 postoperatively (*P* < 0.001 and *P* < 0.001, respectively). The time to first opioid demand was significantly shorter in the control group than in the PENG group (*P* < 0.001). Sensory block effectiveness was better in the PENG group 30 min postblock and 6 and 12 h postoperatively than in the control group. Patient satisfaction was also better in the PENG group than in the control group. There were no differences in the other outcomes. The PENG block reduced the total opioid consumption in the first 24 h after hip surgery with no significant effects on quadriceps muscle strength and complication rate. This study was registered at the Korea Clinical Research Information Service (cris.nih.go.kr; Reg. No. KCT0006348) on July 16, 2021.

## 1. Introduction

A hip fracture is an orthopedic emergency with a high mortality rate [1]. In patients with hip fractures, perioperative pain control is significantly important as inadequately controlled pain can impair early rehabilitation and functional recovery and reduce patient satisfaction after surgery [2, 3]. In addition, effective perioperative analgesia minimizes the need for intravenous opioid use and thereby reduces the incidence of opioid-related adverse effects [4, 5]. Ultrasound-guided peripheral nerve blocks for hip surgery, such as the femoral nerve block (FNB), fascia iliaca compartment block (FICB), and lumbar plexus block (LPB), have been widely used as they provide pain relief and reduce the use of opioid and nonopioid analgesics, thus decreasing the rate of analgesia-related side effects [6, 7]. However, these peripheral nerve blocks have disadvantages in terms of motor weakness of the quadriceps, which may result in a prolonged hospital stay and the length of rehabilitation.

The pericapsular nerve group (PENG) block is a relatively recently described nerve block technique targeting the nerves innervating the anterior surface of the hip joint that has excellent analgesic effects and no quadriceps weakness [1]. The anterior hip joint capsule is innervated by the obturator, accessory obturator, and femoral nerves [1, 8, 9]. Therefore, Girón-Arango et al. [1] suggested that these nerves should be the main targets for hip analgesia that can be expected to reduce pain and subsequent side effects after a hip surgery.

There are currently several case reports and randomized controlled clinical trials of PENG [1, 10–14]. Aliste et al. [10] say that the PENG block results in better preservation of motor function than FICB. Lin et al. [11] report that quadriceps strength was better preserved with the PENG block. In addition, there is clinical research the PENG block is suited for more complete hip analgesia compared with FICB [12]. However, although each of these studies showed the effect of PENG block, they say that further randomized controlled trials using a larger sample size are needed to prove its clinical significance. This study aimed to investigate the effects of the PENG block for hip surgery on postoperative opioid consumption and related side effects, as well as on quadriceps muscle strength. We hypothesized that total opioid consumption would be lower in the group receiving PENG block up to 24 hours postoperatively, and quadriceps weakness would not be different compared to the placebo group. Thus, we determined the effectiveness of the PENG block for hip surgery.

## 2. Materials and Methods

This was a randomized, double-blind, placebo-controlled study conducted at a single institution. This study was approved by the Institutional Review Board (IRB No. DAUHIRB-21-129, approved on June 23, 2021) and registered at the Korea Clinical Research Information Service in cris.nih.go.kr (KCT0006348) on July 16, 2021, prior to patient recruitment. All patients were informed about the anesthesia and surgery procedures and provided written informed consent before enrollment. This study was conducted in accordance with the tenets of the Helsinki Declaration and is reported following the Consolidated Standards for Reporting Trials guidelines. In reporting this study, we adhered to the CONSORT 2010 checklist recommendations.

The study population included patients aged 30–90 years who were scheduled to undergo hip surgery under general anesthesia at our hospital from July 2021 to January 2022, who had an American Society of Anesthesiologists physical status class I, II, or III, and who had agreed to receive a PENG block. All patients did not receive any other rescue blocks while waiting for surgery. The exclusion criteria were as follows: an infection in the area where the block is performed, chronic opioid dependence, morbid obesity (body mass index > 35 kg/m^2^), neurological deficits, bleeding tendency, allergy to ropivacaine (HANLIM Pharmaceutical, Seoul, Korea), poorly controlled diabetes, pregnancy, psychosis, and a lack of consent.

Patients were randomly allocated to receive a PENG block with either 25 mL of 0.5% ropivacaine (PENG group) or 25 mL of isotonic saline solution (control group) before surgery. We used computer-generated randomization with 1 : 1 intergroup ratio. The random assignment table was prepared using the nQuery Advisor® version 7.0 (Statsols, BMDP Statistical Software Inc., Cork, Ireland), and the size of the block was randomly selected. Sequentially numbered, sealed envelopes were prepared. A study assistant, not involved in the anesthesia of study participants, would open the sealed envelope containing group allocation and prepare 25 mL of 0.5% ropivacaine or 25 mL of isotonic saline solution. Another assistant would double-check the procedure. To ensure blinding, one anesthesiologist who was masked for group allocation performed the block, and another anesthesiologist who did not perform the block assessed the intraoperative and postoperative effects of the block. Intraoperative block effect refers to measuring the effect 30 minutes after the block procedure. During this time, preparations for surgery were made, so it was described as intraoperative. Other investigators who were not involved in the data collection evaluated the block result. Block result means measuring the success and failure of a block based on the effects of the block collected by the investigator after the block was implemented. This final investigator, who was aware of the group allocation but did not participate in the actual block procedure, excluded data that met the PENG block failure criteria and then forwarded the final data to the statistician. Therefore, the only person who was aware of a block failure was the final investigator, who was not involved in the actual block procedure. Investigators, except for the final investigator, patients, care providers, outcome assessors, and the statistician were all blinded to the group allocation. Hip surgery was performed by the same surgical team on all patients. In both groups, patients received light sedation with intravenous midazolam 0.03 mg/kg. Acetaminophen 1 g, ketorolac 30 mg, and dexamethasone 0.1 mg/kg were administered intravenously before surgery as multimodal preemptive analgesia.

**Figure 1:**
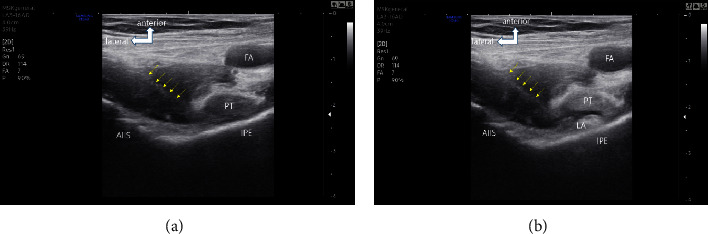
Ultrasound images of pericapsular nerve group block performed via a lateromedial approach. (a) Shows the needle position. The needle is outlined by yellow arrows. (b) Shows the local anesthetic spread following injection. FA, femoral artery; LA, local anesthetic; IPE, iliopubic eminence; AIIS, anterior inferior iliac spine; PT, psoas tendon.

The PENG block was performed with the patients placed in the supine position. A high-frequency linear ultrasound transducer (12–3 MHz; HS40 device; Samsung Medison Ultrasound, Seoul, Republic of Korea) was placed in a transverse plane on the anterior superior iliac spine to identify the anterior inferior iliac spine, iliopubic eminence, and psoas tendon. Using the in-plane technique, the block needle (a 22-gauge Echoplex Plus needle; Vygon Vet. Ltd., UK) was advanced in a lateral-to-medial direction until the needle tip was placed between the iliopubic eminence and psoas tendon. After negative aspiration, 25 mL of 0.5% ropivacaine or saline solution was injected (Figure 1).

The block was always performed by the same anesthesiologists who had performed almost 50 PENG blocks before patient enrollment started. The sensory block was initially assessed 30 min after block administration by pinprick testing using the jagged edges of a broken tongue depressor applied to the skin over the anterior and medial thigh, within the sensory distribution area of the femoral and obturator nerves [13, 15]. Block failure was defined as a case in which there was no decrease in the numerical rating scale (NRS) score by 1 point 30 min postblock and there was no sensory decrease in either the anterior or medial region of the thigh. Repeated block assessment was performed 6, 12, and 24 h postoperatively to determine whether the block was continuous. For each innervation territory, the block was evaluated using a 3-point scale: 0 = no block, 1 = analgesia (the patient can feel touch but not cold), and 2 = anesthesia (the patient cannot feel touch) [16].

All patients were induced by standardized endotracheal general anesthesia with propofol (2–2.5 mg/kg), rocuronium (0.6–2 mg/kg), and fentanyl (0.9 *μ*g/kg) and were maintained with propofol and sevoflurane.

Because the PENG block targets peripheral articular branches, surgical wound infiltration with local anesthetic was necessary. All patients received a wound infiltration of 20 mL ropivacaine 0.375% from the surgeon at the end of the surgical procedure to eliminate any confounding factor linked to the patients' perception of superficial pain.

For postoperative pain management, all patients received the same multimodal analgesia, which included oral acetaminophen 325 mg and tramadol 37.5 mg at 12 h intervals. In addition, intravenous patient-controlled analgesia (IV-PCA) was prepared at a total volume of 100 mL by mixing 20 *μ*g/kg of fentanyl and 0.6 mg of Ramosetron in a saline solution. The baseline infusion rate, bolus demand dose, and lock-out time were 1 mL/h, 1 mL, and 10 min, respectively. All patients were trained on the use of the IV-PCA pump (Accufuser Plus M1015M; Woo Young Medical, Seoul, Republic of Korea) before surgery. The pump was connected at the end of the surgery, and the initial time of IV-PCA was recorded. Opioid consumption was evaluated 24 h after surgery. Patients were instructed to use the pump only if the NRS pain score was greater than 4 points. An additional opioid was administered when the NRS pain score was equal to or greater than 4 points, despite the use of the IV-PCA pump.

The primary outcome was the cumulative IV fentanyl equivalent (IFE) consumption (*μ*g) during the first postoperative 24 h. The remnant volume of the IV-PCA cocktail was measured 24 h after surgery, and the fentanyl dosage used up to that time point was calculated. The IV morphine was converted to IFE.

The secondary outcomes included the following: pain score, time to first opioid demand, sensory block effectiveness 30 min postblock and at 6, 12, and 24 h postoperatively, quadriceps muscle strength at 6, 12, and 24 h postoperatively, postoperative complications, and patient satisfaction. Pain intensity was evaluated using an NRS, with scores ranging from 0 to 10, with 0 indicating the absence of pain and 10 indicating the worst possible pain. Pain scores were recorded before PENG block administration, 30 min postblock, and 6, 12, 18, and 24 h postoperatively. At the time of pain score recording, patients were instructed to perform a straight leg raise of the affected limb to 15°. The time to first opioid demand was defined as the time from arrival at the postanesthesia care unit to the first PCA bolus or other additional opioids, whichever was first, and was categorized every 3 h until postoperative 24 h. Quadriceps muscle strength on the operated side was evaluated before PENG block administration and at 6, 12, and 24 h postoperatively by knee extension testing in the supine position with the patients' hips flexed at 45° and knees flexed at 90°. Patients were instructed to extend the knee first against gravity and then against resistance. Knee extension was graded according to a 3-point scale: 0 = normal strength (extension against gravity and resistance), 1 = paresis (extension against gravity but not against resistance), and 2 = paralysis (no extension possible) [10, 17]. Any complications occurring within 24 h after surgery were recorded. Patients' general satisfaction was classified as satisfied or unsatisfied.

Sample size calculation was performed using the nQuery Advisor® version 7.0 (Statsols, BMDP Statistical Software Inc., Cork, Ireland). The calculation was based on the average total IV fentanyl consumption with mean ± standard deviation (SD) values for hip surgery in the first postoperative 24 h at our hospital over the past year. The retrospective survey of 100 patients after hip surgery revealed a mean ± SD of 630 ± 370 *μ*g during the first 24 hours after surgery. We decided that the minimum relevant reduction of IV fentanyl consumption was 50% (effect size: 0.851) with a level of significance (*α*) = 0.05 (two-sided) and power 80% (1 − *β*) for active versus placebo block. Allowing a 20% drop-out rate, the required sample size was 58 patients in total [18, 19].

All statistical analyses were performed using IBM® SPSS® Statistics software version 26.0 (IBM Corp., Armonk, NY, USA). *P* values less than 0.05 were considered statistically significant. The data are presented frequently with percentages for categorical variables and mean ± SD for continuous variables. Differences in study participants' characteristics were compared across subgroups with the chi-square test or Fisher's exact test for categorical variables and the independent *t*-test or Mann–Whitney's *U* test as appropriate. To check if its distribution is normal, we used Shapiro–Wilk's test. Two-way repeated measures analysis of variance (two-way RM ANOVA) was used to compare repeated measured numeric variables in groups and within each group, and the Bonferroni procedure was applied in post hoc analyses.

## 3. Results

**Figure 2:**
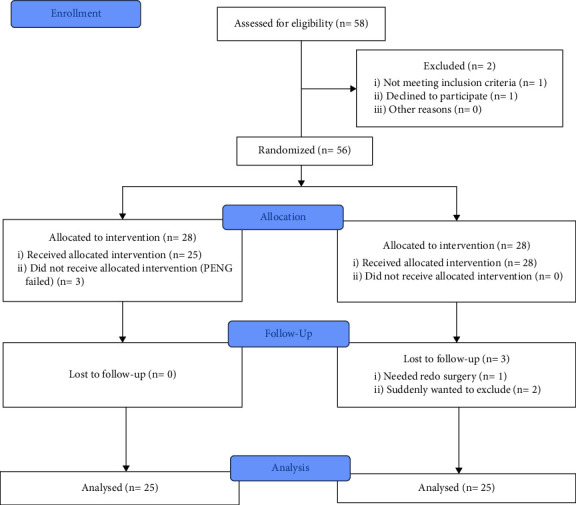
CONSORT flow diagram. CONSORT, consolidated standards of reporting trials; PENG, pericapsular nerve group; control, control group.

During the study period, 58 patients were assessed for eligibility. One patient did not provide informed consent, and one did not meet the inclusion criteria due to previously diagnosed neurological deficits. The remaining 56 patients were randomly and equally allocated between the two groups. Three patients in the control group were lost to follow-up. One of them was required to undergo again the surgery within 24 h postoperatively because of bleeding in the surgical site. The other two patients suddenly wanted to be excluded from this study. Three patients in the PENG group showed no NRS reduction or sensory decrease; thus, PENG block failed. In total, 50 patients completed this study and could be included in the final analysis (Figure 2).

**Figure 3:**
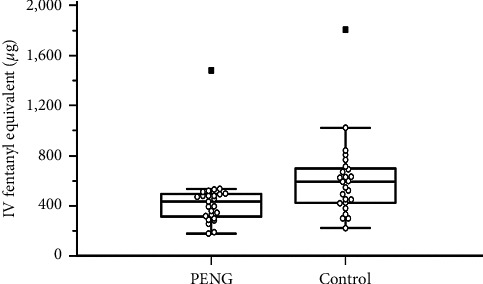
Comparison in intravenous fentanyl equivalent consumption (*μ*g) during the first postoperative 24 hrs, presented as mean ± standard deviation (SD). IV, intravenous; PENG, pericapsular nerve group; control, control group. ^*∗*^A statistically significant difference. *P* values were derived by the independent *t*-test. The Shapiro–Wilk test was employed to test the normality assumption.

There were no clinically relevant differences between the groups in terms of patient's characteristics (Table 1). The IFE consumption in the first postoperative 24 h was significantly lower in the PENG group than in the control group (440.72 ± 242.20 *μ*g vs. 611.07 ± 313.89 *μ*g, *P* = 0.037) (Figure 3 and Table 2).

In a two-way repeated measures ANOVA analysis, NRS significantly decreased during the six assessment periods (*P* < 0.001), and post hoc analyses revealed that this significant change in NRS stemmed from a significant improvement in both groups. This change was different between groups (*P* < 0.001) and the interaction between group and time was also significant (*P* < 0.001), and NRS significantly decreased rapidly from postblock 30 min in the PENG group compared to the control group.

**Figure 4:**
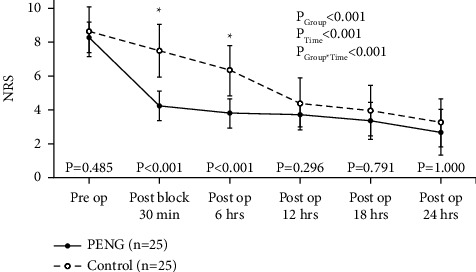
Mean ± standard deviation (SD) plot of pain scores during the first postoperative 24 hrs. NRS, numerical rating scale; PENG, pericapsular nerve group; control, control group; preop, preoperatively; postop, postoperatively. ^*∗*^A statistically significant difference. *P* values were derived from Mann–Whitney's *U* test and adjusted by the Bonferroni correction method. Shapiro–Wilk's test was employed to test the normality assumption.

Additionally, we performed Mann–Whitney test at each assessment timepoint for exploratory analysis, and Bonferroni-adjusted *P* values were presented (Figure 4 and Table 3).

**Figure 5:**
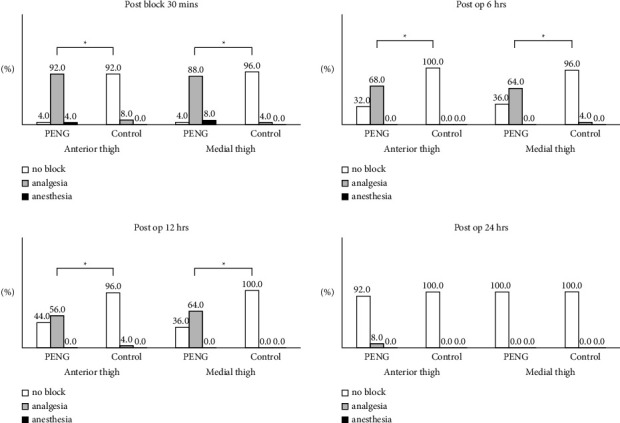
Comparison of sensory block effectiveness between the groups 30 min postblock and 6, 12, and 24 h postoperatively. PENG, pericapsular nerve group; control, control group; postop, postoperatively. ^*∗*^A statistically significant difference. *P* values were derived by the chi-square test.

The time to first opioid demand was significantly different between the two groups, with most patients of the PENG group requiring analgesia after more than 12 h after surgery (Table 4). Statistically significant differences between the groups were also observed in the sensory block effectiveness 30 min postblock and 6 and 12 h postoperatively (*P* < 0.001); however, there was no significant difference at 24 h postoperatively (*P* = 0.490) (Figure 5).

There were no significant intergroup differences in quadriceps muscle strength at any of the assessment time points (Table 4). Patient's satisfaction was significantly greater in the PENG group than in the control group (22 vs. 13, *P* = 0.005).

Regarding postoperative complications, the incidence of hypotension, nausea, vomiting, dizziness, headache, delirium, respiratory depression, and vascular puncture was similar in the two groups. No local anesthetic toxicity or neurological complications were recorded in either group.

## 4. Discussion

In this study, the opioid-sparing effect of the PENG block was less than what we hypothesized. Based on our prior retrospective study, we expected a 50% reduction in IFE consumption in the first 24 h following surgery; however, the observed reduction was lower. This difference remained statistically significant, but not clinically. This is consistent with international efforts to achieve opioid-free or opioid-minimized anesthesia to reduce the risk of addiction and opioid-related side effects due to the long-term use of opioids [17]. The patients in our control group exhibited higher IFE consumption during the 24 h postoperative period, although a multimodal analgesic regimen was applied.

Although we observed a significant decrease in IFE consumption and the NRS pain scores, the majority of patients still required rescue analgesics within 24 h after surgery. Notably, no patients in the PENG group required rescue analgesics during the first postoperative 6 h, compared with 14 patients in the control group. Although this finding is different from that of Del Buono et al. [14], that the PENG block has the best clinical efficacy during the first postoperative 24 h with respect to the duration of the block, we could expect the reduction of total opioid consumption and postoperative complications related to opioid consumption until hospital discharge.

The major targets of the PENG block are the peripheral articular branches of the femoral, obturator, and accessory obturator nerves [1], which dominate the sensory innervation of the medial and anterior aspects of the thigh [13, 15]. Therefore, we divided the thigh into two categories (medial and anterior) and investigated the sensory block's effectiveness at several time points. We found significantly better block effectiveness in the PENG group 30 min postblock and 6 h postoperatively, but not at 24 h postoperatively. Furthermore, statistically, we observed a significant difference in pain score reduction between the groups up to 6 h postoperatively, with a greater pain score reduction in the PENG group than in the control group, although all patients used the IV-PCA pump. The lesser difference in pain score reduction after 6 h postoperatively is due to the fact that bolus usage and total opioid consumption in the control group were greater than those in the PENG group. These may indicate that the duration of the PENG block effect ranges from 6 to 12 h.

The PENG block had no significant effects on the quadriceps muscle strength or incidence of postoperative complications. These findings are consistent with previously reported findings on the PENG block [11, 13]. Anatomical studies have shown that the high branches of the femoral nerve and obturator nerve are distributed in the anterior hip capsule, while the accessory obturator nerve is distributed in the medial hip capsule [9, 10, 20]. Moreover, the highest concentration of nociceptive fibers was located in the anterior and superolateral hip capsules [20]. As the PENG block can most effectively target the nociceptive fibers, it may be an alternative regional analgesia technique to other peripheral blocks, such as the FNB, FICB, and LPB. It also has a potential motor-sparing effect compared to FICB and FNB. This is because it mainly targets the peripheral articular branches [1, 20].

In this study, quadriceps muscle strength in the first 24 h after surgery was intact in the majority of patients in both groups. Preserved quadriceps muscle strength is important because it enables patients to ambulate earlier after hip joint surgery, resulting in reduced pain, fewer complications, and a shorter hospital stay [21–23]. However, we observed a decrease in muscle strength in some patients in the PENG group. Yu et al. [24] reported that superficial local anesthetic injection and needle positioning medial to the psoas tendon may be contributing factors for quadriceps weakness. Some authors have reported a possible obturator motor blockade in the setting of a PENG block if a large anesthetic amount is injected [25]. In fact, we used a larger volume of local anesthetic (25 mL vs. 20 mL) compared with that used by Girón-Arango et al. [1]. As the PENG block is a field block, volume is essential for its success. Yu et al. [24] reported that some of the large local anesthetics may spread superficially and result in an inadvertent FNB or FICB, considering the location of the femoral nerve relative to the psoas tendon. Because we used a larger volume for the PENG block in our study, some patients might have quadriceps weakness. In addition, referring to Figure 5, 92% of the patients in the PENG group experienced a sensory decrease and only 4 percent of the patients experienced a complete sensory block. These 4% of patients may have been caused by FNB. However, there is a limit to prove this in our study. When we evaluated the sensory block, we relied on the subjective feeling that the patient described. In the case of the patients themselves, they didn't know whether or not they were receiving the placebo injection, so in a small percentage of patients, it was thought that the sensory block may have occurred due to the placebo effect.

Furthermore, the PENG block is often a deeper block, which is why visualizing the needle tip during the procedure can be difficult. Therefore, if the needle tip is positioned medially along the iliopubic eminence, an obturator motor blockade may occur [24, 26]. In addition, Acharya and Lamsal [13] reported a technical difficulty with the in-plane approach. That is, when the needle is introduced laterally, the anterior inferior iliac spine comes in the needle path. Thus, the needle needs to be adjusted, which may lead to missed targets [13]. FICB and FNB are superficial blocks performed near the target structure of the PENG block. Therefore, superficial injection of local anesthetics while performing a PENG block may result in inadvertent FICB or FNB, which may explain the presence of quadriceps weakness after surgery [24]. Future studies are required to further investigate this aspect.

In this study, the incidence of postoperative complications, including delirium, respiratory depression, hypotension, nausea, vomiting, headache, dizziness, vascular puncture, and local anesthetic toxicity, was not significantly different between the two groups. This finding corresponds to the results of previous studies [11, 13]. Notably, patients' satisfaction was higher in the PENG group than in the control group.

This study has some limitations. First, we administered a single-shot PENG block for postoperative pain relief. Several trials using catheters to prolong the duration of the block have shown positive results. A meta-analysis comparing the single-shot FNB with the continuous technique has established the excellence of the continuous technique, which increases the duration of analgesia and reduces opioid consumption [27]. Del Buono et al. [14] used catheters to extend the PENG block. They left a catheter in the space between the psoas muscle and the iliopubic eminence for postoperative analgesia. No patient required an additional opioid, and no complications were observed during or after catheter removal. Prado-Kittel et al. [15] also performed a continuous PENG block by placing the catheter between the pubic branch and the tendon of the psoas muscle. They found that the continuous PENG block provided excellent pain relief and extended analgesia to include the distal femoral region. However, both of these publications are case series. Thus, it is necessary to establish the safety and efficacy of the single-shot PENG block and to conduct subsequent studies to clarify its clinical significance. Moreover, Lee et al. [28] reported that the addition of a low dose of naloxone to 0.375% ropivacaine for FNB prolonged the duration of the time to the first request for rescue analgesia and reduced the amount of supplementary opioids consumed without any significant adverse effects. Therefore, we need to study how to prolong the duration of a single block using ropivacaine with added adjuvants and what adjuvants can be added to ropivacaine to extend the duration of the block. Second, we only investigated patients during the first postoperative 24 h. Wylde et al. [29] reported that major hip procedures were accompanied by severe pain, particularly within the first 48 h. Further studies with long-term patient follow-up are required to investigate the effects of the PENG block in relation to chronic pain development. Third, because IV-PCA has a lock-out time, it was not possible to press whenever the patients wanted, and through preinstructing, patients were instructed to press only when the NRS score was 4 or higher. If it was possible to press at the patients' demand without such instruction or lock-out time, there may have been differences in total consumption. However, the side effects of opioid overuse should be considered, and both groups had to be assessed at the same time interval because they could not be assessed at every moment, 24 h a day. Moreover, if the pain was not controlled with PCA alone, additional analgesics were administered as necessary and calculated as IFE.

## 5. Conclusions

In conclusion, the PENG block with 25 mL of 0.5% ropivacaine significantly decreases opioid consumption during the first postoperative 24 h and reduces pain scores during the first postoperative 6 h while having no significant effects on the quadriceps muscle motor strength. For these reasons, it can be considered for pain management in hip surgery.

## Figures and Tables

**Table 1:** Patients' characteristics. Values are presented as *n* (%) or mean ± standard deviation.

Variable	Group		*P*
	PENG (*n* = 25)	Control (*n* = 25)	
Height (cm)	158.52 ± 6.69	159.96 ± 7.74	0.485^1^
Weight (kg)	56.08 ± 8.46	55.43 ± 10.93	0.815^1^
BMI (kg/m^2^)	22.33 ± 3.21	21.26 ± 3.69	0.281^1^
Sex			
Female	14 (56.0)	13 (52.0)	0.777^3^
Male	11 (44.0)	12 (48.0)	
Age (yr)	75.96 ± 7.54	72.60 ± 9.66	0.177^1^
ASA score			
2	13 (52.0)	10 (40.0)	0.395^3^
3	12 (48.0)	15 (60.0)	
Cardiac parameters preblock			
Systolic blood pressure (mmHg)	143.08 ± 22.76	143.04 ± 20.47	0.995^1^
Heart rate (beats/min)	83.32 ± 12.65	92.28 ± 20.69	0.225^2^
Cardiac parameters 30 min postblock			
Systolic blood pressure (mmHg)	128.84 ± 22.25	126.88 ± 22.86	0.760^1^
Heart rate (beats/min)	80.04 ± 13.47	88.64 ± 21.11	0.092^1^
Diagnosis			
ONFH	2 (8.0)	4 (16.0)	0.523^4^
Intertrochanteric fx.	10 (40.0)	7 (28.0)	
Femoral neck fx.	13 (52.0)	14 (56.0)	
Surgery type			
THA	3 (12.0)	6 (24.0)	0.654^4^
CR & TFNA insertion	12 (48.0)	10 (40.0)	
Bipolar hip arthroplasty	10 (40.0)	9 (36.0)	
Anesthesia time (min)	112.60 ± 24.46	112.00 ± 38.62	0.534^2^
Operation time (min)	68.08 ± 20.85	72.60 ± 35.09	0.938^2^
Performance time (min)	5.04 ± 0.89	4.72 ± 0.98	0.246^2^

^1^ *P* values were derived from the independent *t*-test. ^2^*P* values were derived from the Mann–Whitney *U* test. ^3^*P* values were derived using the chi-squared test. ^4^*P* values were derived from Fisher's exact test. PENG, pericapsular nerve group; control, control group; BMI, body mass index; ASA, American Society of Anesthesiologists; ONFH, osteonecrosis of the femoral head; fx, fracture; THA, total hip arthroplasty; CR, closed reduction; TFNA, trochanteric fixation nail-advanced.

**Table 2:** Comparison in intravenous fentanyl equivalent consumption (*μ*g) during the first postoperative 24 hrs. Values are presented as mean ± standard deviation.

Variable	PENG (*n* = 25)	Control (*n* = 25)	*P*
Mean (95% CI)	440.72 ± 242.20	611.07 ± 313.89	0.037^*∗*^
Min	177	222.5	
First quartile	309.6	423.7	
Median	435	594	
Third quartile	467	705.5	
Max	1482.5	1807.3	

^*∗*^A statistically significant difference. *P* values were derived by the independent *t*-test. The Shapiro–Wilk test was employed to test the normality assumption. PENG, pericapsular nerve group; control, control group.

**Table 3:** NRS at each assessment point by the group during the first postoperative 24 hrs. Values are presented as mean ± standard deviation.

Variable	Group		*P* ^2^	RM ANOVA	*P* ^3^
	PENG (*n* = 25)	Control (*n* = 25)		Source	
NRS					
Preop	8.28 ± 0.89a^1^	8.60 ± 1.47a	0.485	Group	<0.001
Postblock 30 min	4.24 ± 0.88b	7.48 ± 1.56b	<0.001	Time	<0.001
Postop 6 hrs	3.80 ± 0.87bc	6.32 ± 1.49c	<0.001	Group × time	<0.001
Postop 12 hrs	3.72 ± 0.74c	4.36 ± 1.55d	0.296		
Postop 18 hrs	3.36 ± 1.08cd	3.96 ± 1.49de	0.791		
Postop 24 hrs	2.68 ± 1.35d	3.24 ± 1.42e	1.000		

^1^Values are the mean ± SD and Bonferroni's post hoc test was used for multiple comparisons between each of the six-time points. That means different scripts are different from each other (*P* < 0.05). ^2^*P* values were derived by Mann–Whitney's *U* test and adjusted by the Bonferroni correction method. Shapiro–Wilk's test was employed to test the normality assumption. ^3^*P* values are derived from an ANOVA with repeated measures with a Greenhouse-Geisser correction used when sphericity was not assumed. RM ANOVA, repeated measures analysis of variance; PENG, pericapsular nerve group; control, control group; NRS, numerical rating scale; preop, preoperatively; postop, postoperatively.

**Table 4:** Secondary outcomes. Values are presented as *n* (%) or mean ± standard deviation.

Variable	Group		*P*
	PENG (*n* = 25)	Control (*n* = 25)	
Time to first opioid demand			
0–3 h postoperatively	0 (0.0)	6 (24.0)	<0.001^1^
3–6 h postoperatively	0 (0.0)	8 (32.0)	
6–9 h postoperatively	3 (12.0)	10 (40.0)	
9–12 h postoperatively	5 (20.0)	1 (4.0)	
>12 h postoperatively	17 (68.0)	0 (0.0)	
Quadriceps muscle strength			
Before block			
0 = intact	20 (80.0)	15 (60.0)	0.113^1^
1 = reduced	5 (20.0)	6 (24.0)	
2 = absent	0 (0.0)	4 (16.0)	
3 = uncheckable			
At 6 h postoperatively			
0 = intact	16 (64.0)	14 (56.0)	0.262^1^
1 = reduced	7 (28.0)	6 (24.0)	
2 = absent	2 (8.0)	1 (4.0)	
3 = uncheckable	0 (0.0)	4 (16.0)	
At 12 h postoperatively			
0 = intact	18 (72.0)	15 (60.0)	0.180^1^
1 = reduced	7 (28.0)	6 (24.0)	
2 = absent	0 (0.0)	4 (16.0)	
3 = uncheckable			
At 24 h postoperatively			
0 = intact	20 (80.0)	15 (60.0)	0.113^1^
1 = reduced	5 (20.0)	6 (24.0)	
2 = absent	0 (0.0)	4 (16.0)	
3 = uncheckable			
Postoperative complications			
Delirium	0 (0.0)	2 (8.0)	0.490^1^
Respiratory depression (hypoxia, hypopnea)	0 (0.0)	0 (0.0)	
Hypotension	0 (0.0)	1 (4.0)	1.000^1^
Nausea	1 (4.0)	1 (4.0)	1.000^1^
Vomiting	1 (4.0)	1 (4.0)	1.000^1^
Headache	1 (4.0)	2 (8.0)	1.000^1^
Dizziness	1 (4.0)	4 (16.0)	0.349^1^
Patient satisfaction			
Satisfied	22 (88.0)	13 (52.0)	0.005^2^
Unsatisfied	3 (12.0)	12 (48.0)	

^1^ *P* values were derived from Fisher's exact test. ^2^*P* values were derived using the chi-squared test. PENG, pericapsular nerve group; control, control group.

## Data Availability

It is being stored on the hospital's data server. Due to personal information issues, it cannot be provided collectively. The authors will provide it separately if there is a later request.

## Supplementary Materials

Supplementary Materials The supplementary material for this article is included within the supplemental file.
